# Therapeutic Notes

**Published:** 1900-11

**Authors:** 

**Affiliations:** Buffalo, N. Y.


### Progress in Medical Science.

### THERAPEUTIC NOTES.

EDITED BY NELSON W. WILSON, M. D., Buffalo, N. Y.

### FOR DELAYED RESOLUTION IN PNEUMONIA.

VERY often in pneumonia one finds resolution rather backward. In such cases the following treatment, Dr. DaCosta's method of procedure, will be found to be of great benefit and in the majority of cases will bring about a splendid result. First apply small blisters over the affected area, and give the following:

| | | | |
|---|---|---|---|
| ℞ | Iodide of potassium | 4.00 | ʒi. |
| | Muriate of ammonia | 6.00 | ʒiss. |
| | Compound licorice mixture | 180.00 | ℥vi. |

Mix. Dose, one tablespoonful four times a day.

---

### PROSTATIC HYPERTROPHY.

The following, which is recommended by the *Revue Médicale*, has been used in three cases of prostatic hypertrophy. In one case it gave no benefit whatever. In the other cases there was apparent relief almost immediately. The case in which failure is recorded was one of long standing.

| | | | |
|---|---|---|---|
| ℞ | Sulficthyolate of ammonia | .33 to .78 | gr. v. to xii. |
| | Cocoa butter | 3.59 | gr. liv. |

Make into one suppository.

---

### FOR PULMONARY TUBERCULOSIS NIGHT SWEATS.

This formula, used as a lotion, is reported by Kohnstamm as being very efficient in the night sweats of pulmonary tuberculosis:

| | | |
|---|---|---|
| ℞ | Balsam of peru | 1 part. |
| | Formic acid | 5 parts. |
| | Chloral hydrate | 5 parts. |
| | Trichloracetic acid | 1 part. |
| | Absolute alcohol | 100 parts. |

---

### DIARRHEA IN CHILDREN.

Dr. C. G. Keeley, in treating diarrhea in children confines himself to the use of castor oil, calomel, bismuth subnitrat and opium. He withdraws milk diet almost wholly and gives, in ordinary cases:

| | | | |
|---|---|---|---|
| ℞ | Subnitrat of bismuth | 1.00 | gr. xv. |
| | Aromatic syrup of rhubarb | .18 | m iii. |
| | Water to make | 4.00 | ʒ i. |

For one dose. Repeat every hour.

---

### FOR RINGWORM.

Two very serviceable prescriptions for ringworm are the following. This from the *Jour. de Médicine de Bordeaux*, has been very successfully used:

| | | | |
|---|---|---|---|
| ℞ | Epicarin | 4.00 | ʒi. |
| | Venetian talc | 4.00 | ʒi. |
| | Starch | 4.00 | ʒi. |
| | Vaseline | 12.00 | ʒiii. |

Make into ointment and use externally.

From the Canada *Medical Record* is this. In some cases it may be made stronger.

| | | | |
|---|---|---|---|
| ℞ | Salicylic acid . . . . . . . . . . . . . . . . | .66 | gr. x. |
| | Vaseline . . . . . . . . . . . . . . . . . . | 4.00 | ʒi |

Make into ointment and apply twice daily.

### FOR FISSURED NIPPLES.

One of the best prescriptions which can be used for this condition is given in a recent number of the Canada *Medical Record:*

| | | | |
|---|---|---|---|
| ℞ | Compound tincture of benzoin . . . . . . . | 8.00 | ʒii. |
| | Listerine . . . . . . . . . . . . . . . . . . | 16.co | ʒiv. |
| | Camphor water, to make . . . . . . . . . | 90.00 | ℥iii. |

Directions—Bathe nipples and apply freely after each nursing. Shake. Label.

### FOR SMALL CUTANEOUS GROWTHS.

Unna's treatment for small cutaneous growths is simple. As reported by the *Medical News*, Unna gives this prescription to the patient who makes frequent applications to the growth:

| | | | |
|---|---|---|---|
| ℞ | Paraformaldehyde . . . . . . . . . . . . | 1.00 | gr. xv. |
| | Castor oil . . . . . . . . . . . . . . . . . | .52 | m viii. |
| | Collodion . . . . . . . . . . . . . . . . | 20.00 | ʒv. |

### TO ABORT BOILS.

Dr. Ochme, in the *Medical World*, covers the affected area freely with collodion, containing from .03 to .13 (gr. ss—ii.) of salicylic acid to the dram. This application is repeated two or three times in twelve hours.

Another abortive method which is used with almost miraculous results is the application of electricity to the area, after painting freely with colorless tincture of iodine. This is particularly desirable where abscess formations are present on the face.

### ENURESIS.

Dr. M. G. Price in the Canada *Medical Record* has by using the following, in a majority of cases, succeeded in overcoming this most rebellious of troubles:

| | | | |
|---|---|---|---|
| ℞ | Tincture of belladonna . . . . . . . . . . . | .33 | gtt. v. |
| | Fluid extract of ergot . . . . . . . . . . . . | .66 | gtt. x. |
| | Tincture of nux vomica . . . . . . . . . . . | .33 | gtt. v. |

Mix for one dose; repeat four times a day.

### NERVE TONIC.

The St. Louis *Clinique* highly recommends the following as a most efficient nerve tonic:

| | | | |
|---|---|---|---|
| ℞ | Asafetida | 4.00 | ʒi. |
| | Arsenious acid | .03 | gr. ss. |
| | Strychnine sulfate | .03 | gr. ss. |
| | Ext. sumbul | 2.00 | ℈ iss. |
| | Subcarbonate of iron | 2.66 | ℈ ii. |
| | Valerianate of quinine | 1.33 | ℈ i. |

Mix and make into 24 capsules.

Dose—One capsule after each meal.

---

### MYALGIA.

This affection in unusually trivial as to duration and consequences, but often is such a source of discomfort to the patient that a ready remedy is welcome:

| | | | |
|---|---|---|---|
| ℞ | Fluid extract cimicifuga. | | |
| | Fluid extract coca. | | |
| | Ammoniated tincture of guiac, of each, | 4.00 | ʒi. |

Dose—A teaspoonful three times a day.

—*Jour. Am. Med. Association.*

---

### POWDERS FOR TRIGEMINAL NEURALGIA.

The *Riforma Medica* gives the following formula:

| | | |
|---|---|---|
| ℞ | Extract of cannabis indica | 7½ grs. |
| | Salicylic acid, | 75 grs. |

Divide into ten powders. One to be taken three times a day.

---

The opposition to the use of antipyretic drugs in fevers seems to be diminishing. O. Rosenbach (*Brit. Med. Jour.*) advises phenacetin or antipyrin (0.25 gm, not more than 0.50 in one day) six or eight hours before the expected rise of temperature in typhoid.

---

Marfan (*Jour. de Med. de Paris*) considers lactation a contra-indication to the following drugs: Opiates, atropin, hyoscyamus, colchicum, arsenic, cocain, chloral, lead salts, which are precipitated by milk, digitalis, ergot, antipyrin, the last three only relatively.

---

A. Napier claims that 50 per cent. of sheep's thyroids are histologically abnormal. While it may be doubted if so large a proportion of American flocks are diseased, and while minor abnormalities may have no therapeutic significance, it is worth while to bear in mind the need of caution.

---

G. Frank Lydston (*Ther. Gaz.*) states his experience with santonin in epilepsy. He does not claim permanent cures, but a marked control of convulsive attacks, and considers santonin far superior to the bromides. He begins with 2 to 5 gr. and increases to about 20 gr., three times a day. He watches the urinary secretion for evidences of overdose.

---

FOR A TICKLING COUGH.

One very often meets with an annoying condition wherein the patient complains of a "tickling in the throat which makes me cough constantly." This will be relieved by the following:

| | | | |
|---|---|---|---|
| ℞ | Codeine . . . . . . . . . . . . . . . . . . | .065 | gr. i. |
| | Phenacetine . . . . . . . . . . . . . | .78 | gr. xii. |
| | Powdered licorice . . . . . . . . . . . . | 1.065 | gr. xvi. |
| | Sugar of milk, a sufficient quantity. | | |

Make into 8 powders. Dose—Dissolve one in mouth every hour.

